# Combinations of mutations in the raffinose synthase genes and the fatty acid desaturase genes for improvement of soybean oil and meal traits

**DOI:** 10.1007/s11032-026-01636-x

**Published:** 2026-01-23

**Authors:** Kristin Whitney, Militza Carrero-Colón, Senay Simsek, Karen Hudson

**Affiliations:** 1 Department of Food Science, Whistler Center for Carbohydrate Research, Purdue University, West Lafayette, IN USA; 2 USDA-ARS Crop Production and Pest Control Research Unit, West Lafayette, IN 47907 USA

**Keywords:** High oleic soybean, Raffinose, Stachyose, Soybean seed composition

## Abstract

Soybean (*Glycine max*) meal and oil are important agricultural products, with soybean oil accounting for 55% of US vegetable oil consumption and soybean meal for 69% of global protein meal consumption. The ideal soybean oil profile for food applications is an oil high in the monounsaturated oleic acid, yet low in polyunsaturated fat which may result in instability and off-flavors. Soybean seeds also produce a limited amount of raffinose and stachyose, which are not digestible by non-ruminant animals, and limit the uses of soybean in meal and other food products. In recent years, efforts in soybean genetics have identified gene variants that can achieve the ideal oil profile and limit the biosynthesis of raffinose and stachyose in the seed. In this work conventional breeding techniques were employed to combine mutant alleles of genes for altered fatty acid and carbohydrate content, and the resulting lines were evaluated for additivity on aspects of seed composition. We found that we could obtain high oleic (> 75%) soybeans with < 2% of the seed mass comprising raffinose and stachyose using a combination of four mutant alleles. High oleic-low linolenic soybean with less than 3% linolenic acid, > 75% oleic acid and raffinose and stachyose < 2% of the seed weight were obtained by combining six genes. Composition changes were consistent over three growing seasons and no detrimental effects on overall seed protein or oil content were observed.

**Supplementary Information:**

The online version contains supplementary material available at 10.1007/s11032-026-01636-x.

## Introduction

The seed composition of soybean is remarkably plastic, permitting modifications to the protein, amino acid, lipid, and carbohydrate content to make the seed a useful ingredient for many different end uses in food and fuel applications. Soybeans are rich in complete protein, with a seed protein content of 40% or more, but oil also comprises a generous 20% of the seed. In the 2022–2023 marketing year, 378 million metric tons of soybean was produced globally (with the US producing over one quarter of this crop) making it the largest oilseed crop in the world (Soystats 2024). Following post-harvest processing, the seed oils find uses in industrial and food applications, while the soybean seed meal serves as a rich source of protein for animal (including poultry, swine, and dairy) feed and food industry uses. Carbohydrates make up ~ 35% of the soybean seed, and these include the relatively digestible sucrose, which varies from 1–10% across soybean germplasm accessions (Qiu et al. 2015). Other soluble carbohydrates in soybean seed include the oligosaccharides raffinose and stachyose, collectively termed raffinose family oligosaccharides (RFOs). Stachyose levels across soybean genotypes range from 1–4%, and raffinose content ranges from 0.1–1% (Hymowitz et al. 1972). While present at relatively low levels in the seed, these RFOs are not digestible by monogastric animals, including humans, and when consumed are broken down by gut microflora resulting in flatulence (Liener 1994). Soybean meal with reduced levels of raffinose and stachyose has been shown to have improved digestibility and has higher metabolizable energy than standard soybean meal for a variety of animals including pig, chicken, and dogs (Karr-Lilienthal et al. 2005; Parsons et al. 2000). Soybean breeding efforts have explored low- and ultralow-RFO traits to reduce these antinutritional compounds, and while primarily advantageous for animal feeding applications, this trait may expand opportunities for food grade soybeans and soy food products (Singer et al. 2023; Deak et al. 2006). Biotechnological approaches, such as silencing the *RAFFINOSE SYNTHASE2* (*RS2*) (Glyma.06g179200) gene have been shown to increase sucrose while decreasing the amount of raffinose and stachyose in the seed (Valentine et al. 2017). Induced and naturally occurring mutations in the *RS2* gene have been shown to have the same effect (Dierking and Bilyeu 2008, 2009a, b; Silva et al. 2019; Skoneczka et al. 2009; Bilyeu and Wiebold 2016). Mutation in the homologous *RAFFINOSE SYNTHASE3* (*RS3*) (Glyma.05g003900) further reduces stachyose and raffinose when combined with the *rs2* mutation (Hagely et al. 2020; Jo et al. 2019; Thapa et al. 2019). Other genes in the raffinose and stachyose biosynthesis pathway have also been shown to also affect sucrose, stachyose, and raffinose levels including the genes for stachyose synthase and galactinol synthase (Qiu et al. 2015; Le et al. 2020). RFO accumulation in plant tissues is associated with stress conditions, and RFOs have a role in seed dessication tolerance (Kumar et al. 2010; Peterbauer and Richter 2001). The reduction in RFO content in soybean by the *rs2* mutation was found to not interfere with germination (Dierking and Bilyeu 2008) Recent research highlights the functional properties of RFOs in animal and human diets, particulary through their impacts on the gut microbiome (reviewed recently in Elango et al. 2022).

Soybean oil, at 28% of global oil consumption, is the second most-consumed vegetable oil worldwide (Soystats 2024). Commodity soybean seed oil is a mixture of five major fatty acids: palmitic acid (typically making up 10% of the oil fraction), stearic acid (4%), oleic acid (22%), linoleic acid (56%) and linolenic acid (8%) (Hammond and Fehr 1984). Genetic efforts in soybean breeding have targeted the reduction of the polyunsaturated fats linoleic and linolenic acid which reduce the functionality of the oil through negative impacts on flavor and stability (Fehr 2007). Loss-of-function mutations in the omega-6 fatty acid desaturase (*FAD2*) genes (Glyma.10g278000 and Glyma.20g111000) impair the enzymatic step that produces linoleic acid and singly result in elevated levels of oleic acid up to 40% of the total oil content, but in combination can confer levels of up to 75% oleic acid in the seed (Pham et al. 2010, 2011; Sweeney et al. 2017). High oleic (HO) soybean oil resulting from these genetic changes (whether naturally occurring, induced, or modified through biotechnological approaches) is desirable for food uses. The omega-3 fatty acid desaturase genes Glyma.14g194300 (*FAD3A*) and Glyma.18g062000 (*FAD3C*) further reduce levels of linolenic acid to as little as 2% when deployed in combination with *fad2* mutations (Bilyeu et al. 2003; Heppard et al. 1996; Reinprecht and Pauls 2016; Pham et al. 2014). The soybean oil profile has been further improved for different downstream uses by combining mutations in the *FAD2* genes with mutations in other fatty acid biosynthesis genes to reduce levels of linolenic acid, to increase or reduce levels of saturated fats (Pham et al. 2012; Bilyeu et al. 2018; Gaskin et al. 2021) and to combine the high oleic trait with elevated vitamin E levels in the seed (Hagely, et al. 2021). HO soybean oil is highly sought to produce healthier vegetable oils, but HO soybeans increasingly have been found to offer benefits in poultry and dairy feed (Baer, et al. 2021; Hanno et al 2024; Toomer et al. 2024).

To enable the breeding of soybean with both added-value meal and oil traits, we have studied the combination of the high oleic and high oleic plus low linolenic fatty acid trait with the ultralow raffinose-family oligosaccharide meal trait. This project explored the effects of the combination of up to six mutant alleles for non-GMO amelioration of seed composition. Here we evaluate the reproducibility and stability of the high oleic, high oleic low linolenic, and the low- and ultra-low RFO seed composition traits in combination over multiple seasons, to determine if these multiple single gene traits can be combined additively.

## Methods

### Plant materials and growth conditions

Plants were grown in the fields at the Purdue Agronomy Center for Research and Education in West Lafayette, Indiana. Single mutant *FAD2* and *FAD3* alleles were originally obtained by screening a chemically-mutagenized population of Williams-82, as part of a forward-genetic screen for variation for seed composition (Hudson 2012; Held et al. 2019; Sweeney et al. 2017; Thapa et al. 2018, 2016a). The *rs3*_*G75E*_ mutant was obtained through a reverse genetic TILLInG approach in the same population and crossed to a line carrying a naturally occurring deletion in the *rs2* gene (*rs2*_*W331-*_) from accession PI200508 as described previously (Thapa et al. 2019; Jo 2016). The HO and LL lines (Held et al. 2019; Sweeney et al. 2017) were combined with the *rs2* and *rs3* alleles by conventional crossing, and one of the HO-*rs2* individuals was crossed to LL-*rs3* in the field. F_1_ plants were advanced in the greenhouse during the winter, and the following season approximately 380 F_2_ plants were genotyped using methods and markers described previously (Sweeney et al. 2017; Thapa et al. 2016a, 2018, 2019; Held et al. 2019; Hudson and Carrero-Colón 2025). No individuals homozygous for all six alleles were recovered, but heterozygotes for the *rs* alleles were propagated to obtain the HOLL-*rs2*, HOLL-*rs3,* HOLL-*rs2rs3*, and control HOLL siblings. The crossing scheme to obtain sextuple mutants is shown in Fig. S1. In each cross, individuals carrying the desired combinations were selected by genotyping and verified by phenotyping at the end of the season. Self-pollinated progeny of these lines were grown and phenotyped in the three subsequent growing seasons for this study. Each subsequent season 10–40% of the individuals in each plot were genotyped to confirm the expected genotypes, and plants were individually harvested for composition analysis. DNA for genotyping was prepared using the Omega EZ plant DNA kit.

### Fatty acid analysis

For seed composition analysis, plants were grown in experimental plots of 20 plants per 6-foot row, with a row spacing of 36 inches, and harvested individually at maturity. Fatty acid content was measured using gas chromatography using a flame ionization detector on bulks of five seeds per plant as described previously (Devereaux et al. 2024; Thapa et al. 2016b). Agilent OpenLab Chemstation software was utilized to run the fatty acid sequence, eliminating myristate and palmitoleate and normalizing the total fatty acids for the five major soybean fatty acids to 100 percent, using the RM-6 standard (Matreya LLC, State College, PA). Data was exported to Microsoft Excel and mean values for fatty acid profiles in Table 1 are presented as a percent of total fatty acid, averaged over three or more individuals as specified. To determine statistical significance of fatty acid differences, analysis of variance and Tukey HSD tests were performed using R software, which was also used to generate plots of the data.

**Table 1:** Seed fatty acids in HO, HOLL, and low RFO lines

	2021						2022						2023					
	Pal	Ste	Ole	Lle	Lln	*n*	Pal	Ste	Ole	Lle	Lln	*n*	Pal	Ste	Ole	Lle	Lln	*n*
W82	11.5 ± 0.3	4.0 ± 0.1	20.6 ± 1.0	57.1 ± 0.8	6.9 ± 0.2	18	10.7 ± 0.2	4.5 ± 0.1	23.2 ± 1.0	54.4 ± 0.8	7.2 ± 0.5	6	10.9 ± 0.1	4.2 ± 0.2	21.6 ± 0.8	56.1 ± 0.6	7.3 ± 0.4	6
HO	6.4 ± 0.1	3.7 ± 0.1	81.0 ± 0.5	4.2 ± 0.3	4.6 ± 0.2	6	6.7 ± 0.9	4.0 ± 0.9	77.6 ± 2.5	6.0 ± 1.7	5.6 ± 0.5	32	6.1 ± 0.3	3.7 ± 0.1	78.7 ± 1.0	6.0 ± 0.6	5.4 ± 0.3	9
HO-*rs2*	6.9 ± 0.2	3.2 ± 0.1	81.2 ± 1.0	4.2 ± 0.3	4.5 ± 0.7	6	6.4 ± 0.4	3.7 ± 0.3	79.4 ± 2.1	5.1 ± 1.2	5.4 ± 0.4	14	7.1 ± 1.5	3.4 ± 0.3	79.7 ± 2.4	4.7 ± 1.6	5.1 ± 0.8	41
HO-*rs3*	7.5 ± 0.3	3.6 ± 0.1	78.9 ± 1.1	5.9 ± 0.9	4.1 ± 0.9	3	7.1 ± 0.1	4.0 ± 0.1	76.5 ± 1.0	6.5 ± 0.7	5.9 ± 0.4	4	7.4 ± 0.7	3.8 ± 0.3	78.1 ± 1.2	5.4 ± 0.6	5.3 ± 0.3	9
HO-*rs2rs3*	7.7 ± 0.3	3.5 ± 0.1	80.3 ± 0.9	4.2 ± 0.7	4.3 ± 0.4	4	8.5 ± 1.8	4.0 ± 0.3	77.6 ± 1.6	4.8 ± 1.0	5.0 ± 0.4	11	7.4 ± 0.4	3.4 ± 0.1	79.5 ± 1.1	5.3 ± 1.3	4.3 ± 0.6	16
HOLL	7.4 ± 0.5	3.6 ± 0.1	81.0 ± 0.6	6.2 ± 0.7	1.9 ± 0.1	12	7.4 ± 0.7	4.1 ± 0.3	78.6 ± 2.0	7.6 ± 1.9	2.3 ± 0.3	7	6.9 ± 0.3	3.5 ± 0.2	79.6 ± 0.9	7.8 ± 0.8	2.2 ± 0.2	39
HOLL-*rs2*	7.3 ± 0.3	3.4 ± 0.2	80.4 ± 0.7	7.0 ± 0.7	2.0 ± 0.1	15	7.0 ± 0.2	3.9 ± 0.2	77.2 ± 1.3	9.3 ± 1.1	2.6 ± 0.2	25	7.0 ± 0.2	3.5 ± 0.1	77.4 ± 1.7	9.7 ± 1.3	2.4 ± 0.2	36
HOLL-*rs3*	7.3 ± 0.2	3.4 ± 0.1	80.6 ± 0.5	6.7 ± 0.4	1.9 ± 0.1	15	7.3 ± 0.3	4.0 ± 0.9	77.3 ± 1.3	9.0 ± 1.0	2.5 ± 0.2	25	7.1 ± 0.2	3.6 ± 0.2	78.4 ± 1.1	8.5 ± 0.8	2.4 ± 0.2	37
HOLL-*rs2rs3*	7.1 ± 0.3	3.4 ± 0.2	80.5 ± 3.0	7.1 ± 2.6	2.0 ± 0.1	21	6.9 ± 0.2	3.6 ± 0.2	77.8 ± 1.6	9.2 ± 1.4	2.5 ± 0.2	64	6.9 ± 0.2	3.4 ± 0.2	78.5 ± 1.5	8.8 ± 1.3	2.3 ± 0.2	94

Fatty acids are expressed as % of total fatty acid, with standard deviations, averaged over *n* samples each year. *Pal* palmitic acid, *Ste* stearic acid, *Ole* oleic acid, *Lle* linoleic acid, and *Lln* linolenic acid

### Seed carbohydrate phenotype determination

Soluble carbohydrates (galactinol, sucrose, raffinose, and stachyose) in soybean were determined by following the method described (Jo et al. 2019) with some modifications. Determination was carried out by high performance ion chromatography with pulsed amperometric detection (PAD) using a Dionex ICS-6000 with an electrochemical detector (Thermo Scientific Dionex). A 10 g sample of seeds from all genotypes were ground in a UDY mill. First, 12.5 mg of sample was extracted with 1 mL of 50% ethanol for 30 min at 70 °C with three times intermittent shaking in a 2-mL microcentrifuge tube. Then, samples were centrifuged 10 min at 16,000 g. Approximately 700 µL of the supernatant was removed and stored at 4 °C before further experiments. A 200-μL aliquot of each sample was dried under vacuum and resuspended in 250 µL of deionized water. Samples were filtered with a 0.45 µm nylon syringe filter and placed in vials before injection. Four soluble carbohydrates (galactinol, sucrose, raffinose, and stachyose) were separated on a Dionex CarboPac PA 1 analytical column connected to a CarboPac PA 1 guard column. The mobile phase was water and 200 mM NaOH with a flow rate of 1.0 mL min^−1^. A gold electrode was used in the electrochemical cell of the detector, and the gold carbo-quad potential was used for detection of sugars. Run time was a total of 70 min, with the first 30 min for sample separation, followed by a 15-min washing step with 200 mM NaOH, and a 15-min re-equilibration step with 90 mM NaOH. Peak areas were integrated for galactinol, sucrose, raffinose, and stachyose. Carbohydrates were quantified based on standard curves generated for each carbohydrate. The contents of galactinol, sucrose, raffinose, and stachyose are reported here as a percentage of seed weight.

### Protein and oil analysis

Seed total protein and oil were measured on bulks of 15 seed from single plants using the mirror cup of the Perten DA 7250 NIR analyzer (Perten, Springfield, IL), replicates represent different plants from the plots (biological replicates) and are expressed as a percentage of dry weight basis. The calibration file is biased annually with soybean seed samples validated with chemical composition analysis. Statistical significance was determined by pairwise two-tailed, type 2 *t*-tests with wild type samples from the same year.

## Results

*FAD* double mutant combinations conferring the most ideal fatty acid profiles from previous work were chosen for combination with the raffinose synthase mutants. Lines carrying the genes conferring the high oleic acid phenotype (*fad2-1a*_W194*_ and *fad2-1b*_*P284S*_) (Sweeney et al. 2017) or the low linolenic acid phenotype (*fad3a*_W81*_ and *fad3c*_*P266S*_) (Held et al. 2019) were combined with mutant alleles responsible for the low RFO trait (*rs2*_*W331-*_ and *rs3*_*G75E*_) (Thapa et al. 2019). Both the *fad2-1a* mutant allele and the *fad3a* mutant allele are nonsense mutations that confer a strong phenotype alone and in combinations. All alleles save *rs2*_*W331-*_ were isolated in the Williams-82 genetic background, allowing an opportunity to compare small effects on phenotypes without significant differences in maturity or differences due to the action of other genes in the background. Appearance and size of seeds of the mutant combinations was not significantly different from the parent line (Fig. S2).

### The high oleic/high oleic low-linolenic fatty acid phenotype is not affected by *rs2rs3*

**Fig. 1:**
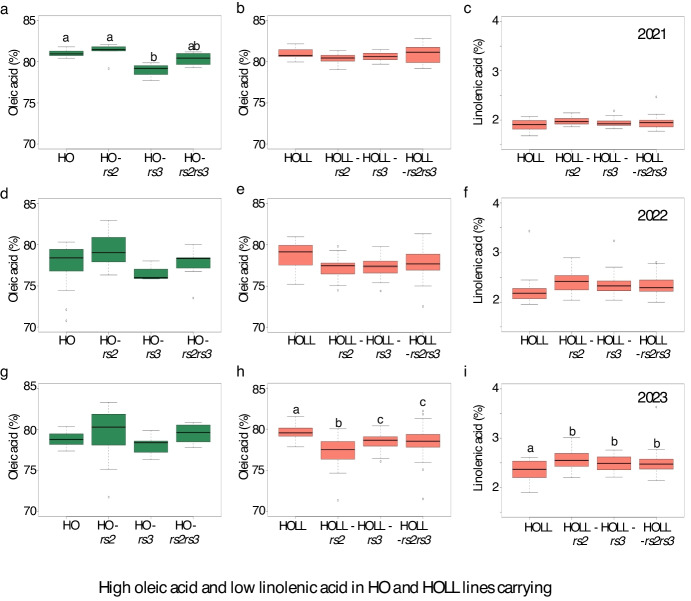
High oleic acid and low linolenic acid in HO and HOLL lines carrying combinations of the *rs2* and *rs3* alleles over three growing seasons. HO lines (**a**, **d**, **g**) carry *fad2-1a* and *fad2-1b* mutant alleles in addition to the *rs2* and *rs3* alleles indicated. HOLL lines (**b**, **c**, **e**, **f**, **h**, **i**) lines carry *fad2-1a*, *fad2-1b*, *fad3a*, and *fad3c* mutant alleles. Fatty acids are expressed as a percent of total fatty acids. Significance by Tukey HSD at *p* < 0.05

We first considered the effects of mutation in the carbohydrate metabolism genes on fatty acid composition phenotypes. All of the lines carrying the *fad2-1a* and *fad2-b* genes averaged more than 77% oleic acid through all three seasons (compared to the wild-type oleic levels of 20–23% oleic acid in the Williams-82 background) (Table 1). Oleic acid levels remained high when the *rs2* allele was present. A small decrease in oleic acid levels were observed in the HO-*rs3* relative to the HO sibling line, but this effect was only statistically significant in 2021 (Fig. 1a). The HO-*rs2rs3* line averaged between 77.6% and 80.3% oleic acid (Fig. 1a, d, and g). We did not observe consistent significant differences in palmitic, stearic, linoleic or linolenic acid in lines carrying the *rs2* or *rs3* alleles (Fig. S3).

The HOLL combinations averaged between 77–81% oleic acid and 1.9–2.6% linolenic acid (Fig. 1). Oleic and linolenic acid levels differed slightly between the *rs2*- and *rs3*- containing lines from the HOLL sibling lines only in the 2023 season, with the *rs2* and *rs3* mutants being and slightly lower in oleic acid and higher in linolenic acid (Fig. 1h, i). No consistently significant changes to the levels of palmitic acid, stearic acid, or linoleic acid were observed (Fig. S4). From these results we conclude that the addition of the *rs2* and *rs3* alleles singly or together does not negatively impact the desired HO or HOLL fatty acid profile.

### The low RFO carbohydrate phenotype conferred by *rs2rs3* is not affected by HO or HOLL

**Fig. 2:**
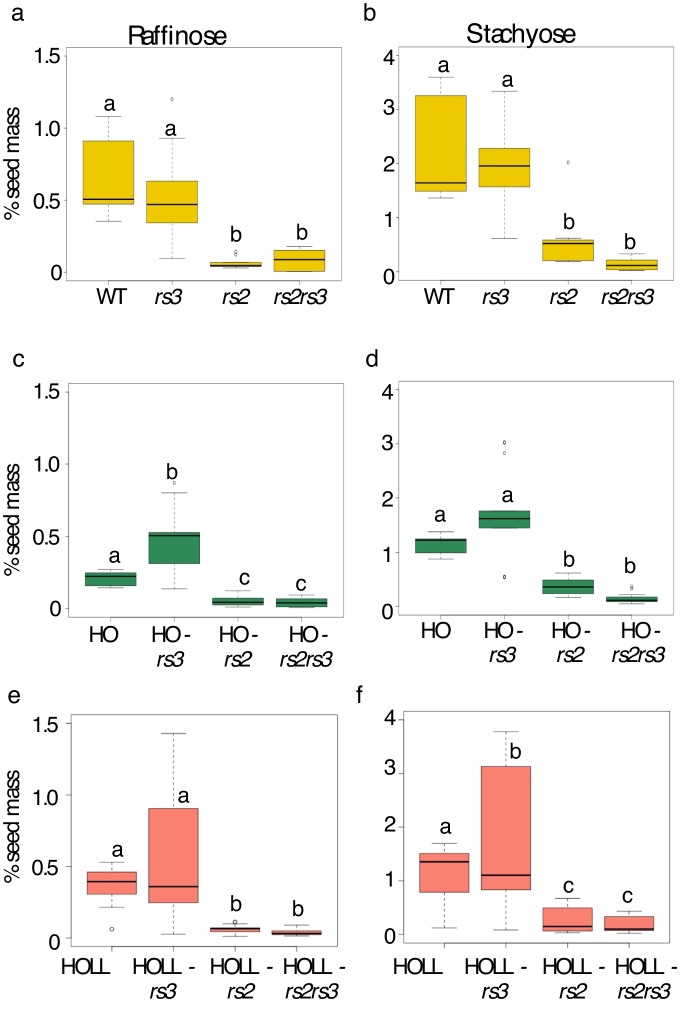
Effect of *rs2* and *rs3* allele combinations on seed RFOs in the wild type (**a**, **b**) HO (**c**, **d**) and HOLL (**e**, **f**) backgrounds. Data is averaged over all three growing seasons. Significance determined by Tukey HSD at *p* < 0.05

Next, we examined how the seed carbohydrate profile is affected by the addition of *rs2*, *rs3* or *rs2rs3* to the HO and HOLL lines. Raffinose ranged from 0.5% to 1% of total carbohydrates in Williams-82. Interestingly, the Williams82 wild type had slightly higher levels of raffinose and stachyose than the HO or HOLL lines (Table 2). Figure 2 confirms that the presence of the mutant allele of *rs3* alone does not significantly reduce levels of raffinose or stachyose in either the wild type, HO or HOLL background, as previously observed (Hagely et al. 2020; Thapa et al. 2019; Kim et al. 2024) while the *rs2* allele alone causes a significant reduction in levels of raffinose (by 90%) and stachyose levels (by 30–90%) in HO and HOLL. The combination of *rs2rs3* provides a significant reduction in raffinose and stachyose from HO or HOLL alone, but not significantly lower than that conferred by the *rs2* mutation in combination with the HO and HOLL traits.

**Table 2:** Carbohydrates in HO- and HOLL lines with the *rs2rs3* alleles

	2021					2022					2023				
	Gal	Suc	Raff	Stach	*n*	Gal	Suc	Raff	Stach	*n*	Gal	Suc	Raff	Stach	*n*
W82	0.01 ± 0.0	2.81 ± 0.0.15	0.99 ± 0.06	3.37 ± 0.13	5	0.44 ± 0.03	3.20 ± 0.06	0.37 ± 0.02	1.67 ± 0.02	3	0.32 ± 0.03	2.70 ± 0.22	0.51 ± 0.04	1.52 ± 0.10	9
HO	nd	nd	nd	nd	nd	0.09 ± 0.01	2.23 ± 0.48	0.21 ± 0.06	1.14 ± 0.21	7					
HO-*rs2*	0.32 ± 0.05	2.21 ± 1.49	0.15 ± 0.12	0.78 ± 0.66	6	1.91 ± 0.75	5.80 ± 1.63	0.02 ± 0.01	0.38 ± 0.17	8	1.39 ± 0.32	3.48 ± 0.18	0.08 ± 0.02	0.43 ± 0.10	8
HO-*rs3*	0.01 ± 0.0	2.71 ± 0.12	0.82 ± 0.05	2.96 ± 0.11	3	0.06 ± 0.0	0.83 ± 0.05	0.15 ± 0.01	0.55 ± 0.01	3	0.52 ± 0.32	2.80 ± 0.18	0.46 ± 0.09	1.62 ± 0.1	8
HO-*rs2rs3*	0.62 ± 0.03	3.25 ± 0.02	0.05 ± 0.01	0.35 ± 0.03	2	1.85 ± 0.79	3.53 ± 0.77	0.01 ± 0.01	0.10 ± 0.03	8	1.11 ± 0.08	2.60 ± 0.48	0.07 ± 0.03	0.13 ± 0.06	9
HOLL	0.25 ± 0.24	1.08 ± 0.35	0.29 ± 0.13	0.71 ± 0.31	9	0.4 ± 0.09	2.19 ± 0.23	0.40 ± 0.07	1.38 ± 0.13	7	0.27 ± 0.02	2.70 ± 0.51	0.46 ± 0.04	1.52 ± 0.14	9
HOLL-*rs2*	0.54 ± 0.05	3.28 ± 0.20	0.08 ± 0.02	0.51 ± 0.07	9	1.27 ± 0.14	2.51 ± 0.60	0.03 ± 0.01	0.05 ± 0.01	6	1.09 ± 0.28	2.28 ± 0.62	0.06 ± 0.03	0.13 ± 0.05	9
HOLL-*rs3*	0.02 ± 0.03	2.87 ± 0.21	1.13 ± 0.19	3.39 ± 0.21	9	0.24 ± 0.08	2.42 ± 0.31	0.40 ± 0.11	1.28 ± 0.36	12	0.29 ± 0.14	1.23 ± 0.23	0.21 ± 0.07	0.73 ± 0.23	9
HOLL-*rs2rs3*	0.52 ± 0.11	3.33 ± 0.33	0.06 ± 0.02	0.36 ± 0.05	9	1.29 ± 0.23	3.19 ± 0.42	0.03 ± 0.01	0.08 ± 0.04	4	0.84 ± 0.08	1.71 ± 0.19	0.02 ± 0.01	0.08 ± 0.02	9
*rs2*	0.26 ± 0.01	0.84 ± 0.04	0.04 ± 0.0	0.19 ± 0.01	3	2.59 ± 0.1	4.03 ± 0.22	0.04 ± 0.01	0.53 ± 0.09	3	1.84 ± 1.14	3.33 ± 0.21	0.11 ± 0.04	1.04 ± 0.85	3
*rs3*	0.07	2.79	1.20	3.34	1	0.50 ± 0.22	2.86 ± 0.76	0.47 ± 0.28	1.76 ± 0.6	6	0.28 ± 0.07	2.99 ± 0.74	0.41 ± 0.19	1.96 ± 0.72	3
*rs2rs3*	nd	nd	nd	nd	nd	1.04 ± 0.80	3.44 ± 1.79	0.02 ± 0.02	0.06 ± 0.04	5	1.47 ± 0.26	3.66 ± 0.22	0.15 ± 0.03	0.22 ± 0.08	5

Carbohydrates are expressed as a percentage of seed weight. Gal = galactinol, Suc = sucrose, Raff = raffinose, Stach = stachyose. *n* = number of individual plants sampled. nd = not determined

**Fig. 3:**
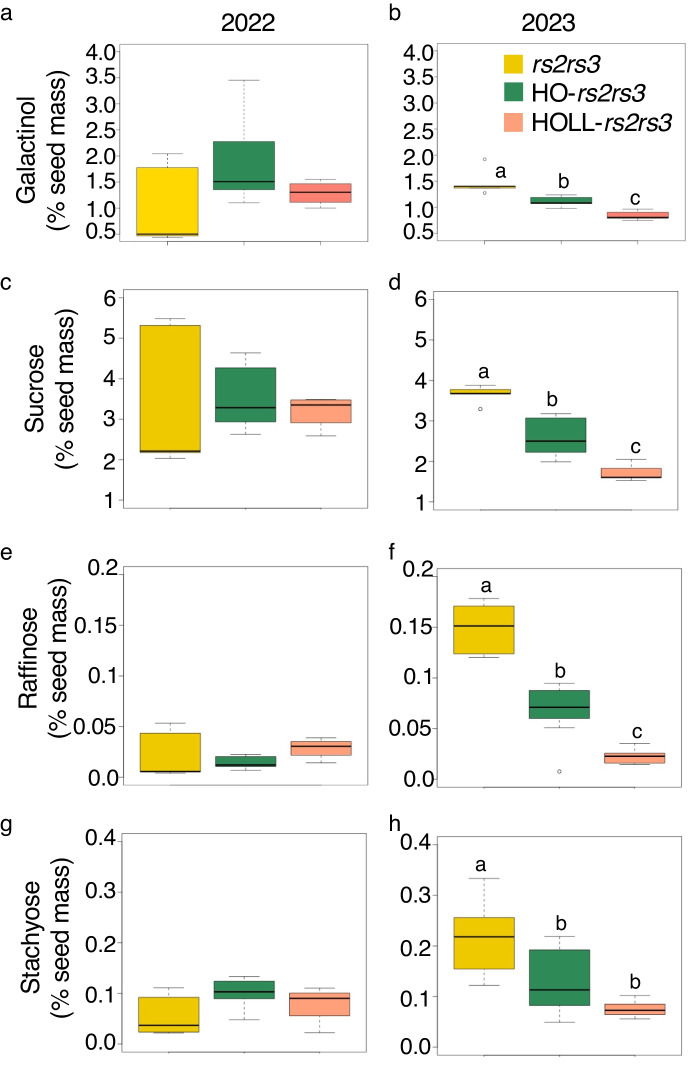
Effect of *rs2rs3* mutations on carbohydrate levels in HO and HOLL background. Galactinol (**a**, **b**) and sucrose (**c**, **d**) is significantly reduced in the HO and HOLL background in 2023. HO or HOLL provide small but significant reduction in raffinose (**f**) or stachyose (**g**) levels in 2023. Significance determined by Tukey HSD at *p* < 0.05

When levels of carbohydrates are compared across *rs2rs3* lines varying for the *fad* alleles in two seasons (Fig. 3) galactinol levels ranged from 0.5–2.0%, sucrose ranged from 2–5%, raffinose levels were below 0.15%, and stachyose levels were below 0.3%. These values did not vary significantly between lines wild type and mutant for HO or HOLL in 2022, but there was a significant reduction in all of the carbohydrates measured in the HO and HOLL lines in 2023 (Fig. 3b, d, f, and h). Therefore, it appears that the fatty acid trait conferred by mutations in the four *fad* genes can be added to the ENEM trait without interfering with the reduction of raffinose and stachyose by *rs2* and *rs3*. Sucrose is significantly reduced by the HOLL alleles with respect to Williams-82 in 2021 and 2022 but not 2023 (Table 2).

### Total seed protein is stable in and * HOLL-rs2rs3*

**Fig. 4:**
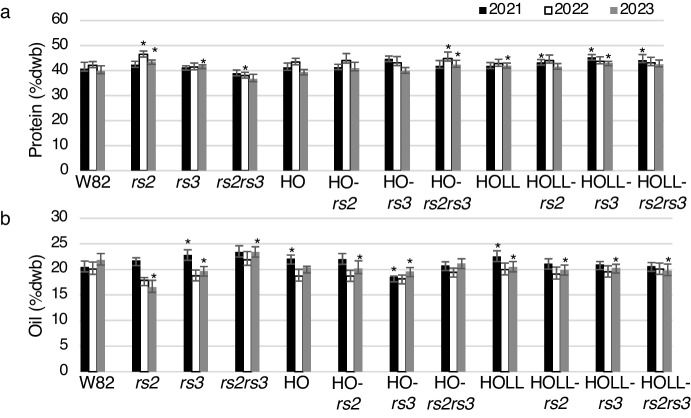
Protein and oil in wild type (W82), HO-, and HOLL- lines carrying *rs2rs3* mutations. Total protein and oil were determined by NIR and expressed as a percentage of dry weight (dwb). Statistical significance was calculated by pairwise t-test each year between the mutant lines and the Williams-82 wild type

We wanted to determine whether the introduction of *rs2rs3* changed levels of protein or oil in the HO or HOLL background (since it is likely that these are the combinations that will be of interest for development.) Overall protein levels varied by only a few percent across all the genotypes (Table 3). Reproducible and consistent differences in total seed protein across years were not observed in the *rs2rs3* lines (Fig. 4a). There was also no trend observed for total seed oil levels between the seeds in the experiment or across growing seasons (Fig. 4b).

**Table 3:** Protein and Oil

	2021			2022			2023		
	Pro	Oil	*n*	Pro	Oil	*n*	Pro	Oil	*n*
W82	40.8 ± 2.5	20.5 ± 1.1	5	42.2 ± 1.4	20.0 ± 1.4	3	39.8 ± 2.1	21.8 ± 1.3	9
HO	41.1 ± 1.9	22.1 ± 0.8	6	43.0 ± 1.1	18.5 ± 1.4	5	39.3 ± 1.1	20.3 ± 0.3	3
HO-*rs2*	41.4 ± 0.8	22.0 ± 1.1	3	43.8 ± 2.4	18.8 ± 1.2	8	40.9 ± 2.4	20.2 ± 1.5	8
HO-*rs3*	44.6 ± 1.2	18.6 ± 0.1	3	42.3 ± 2.6	18.2 ± 0.7	3	39.9 ± 1.2	19.6 ± 0.8	8
HO-*rs2rs3*	41.9 ± 2.1	20.8 ± 0.7	2	45.5 ± 1.7	19.3 ± 0.5	8	42.3 ± 1.7	21.3 ± 0.9	9
HOLL	41.2 ± 1.4	20.2 ± 1.1	9	42.7 ± 1.8	20.0 ± 1.3	7	41.9 ± 1.0	20.5 ± 1.0	9
HOLL-*rs2*	43.2 ± 1.1	21.1 ± 1.0	9	44.0 ± 2.2	19.1 ± 1.4	9	41.5 ± 1.3	19.9 ± 1.0	9
HOLL-*rs3*	45.2 ± 1.1	20.9 ± 0.6	9	43.7 ± 1.8	19.5 ± 1.2	12	43.0 ± 0.7	20.3 ± 0.7	11
HOLL-*rs2rs3*	44.2 ± 2.2	20.6 ± 0.7	9	43.0 ± 2.2	20.1 ± 1.2	7	42.4 ± 1.8	19.8 ± 1.3	9
*rs2*	42.3 ± 1.4	21.8 ± 0.5	5	46.4 ± 1.4	17.8 ± 0.6	3	43.5 ± 0.7	16.5 ± 1.4	3
*rs3*	41.1 ± 0.8	22.8 ± 1.0	5	41.6 ± 1.5	18.9 ± 1.0	6	41.8 ± 0.5	19.7 ± 0.9	3
*rs2rs3*	38.9 ± 1.3	23.4 ± 1.2	10	38.0 ± 1.3	21.8 ± 1.7	5	37.2 ± 1.3	23.2 ± 0.9	6

Protein and oil as a percentage of seed dry weight basis. Average and standard deviation were calculated each year over *n* individuals as listed

## Discussion

Recent progress in breeding soybean for vegetable oil markets has resulted in major positive changes to the fatty acid profile of soybean oil. The soybean crop presents enormous opportunity for value, and work to improve protein levels, amino acid balance, and reduce levels of antinutritional components has been ongoing. Molecular breeding combining these composition traits is well-suited to capturing the soybean’s value to both markets. The breeding of soybean with complex combinations of composition traits is a multi-year process which requires significant investment in reagents and effort for plant selection, genotyping and phenotyping. Seed development is a meticulously orchestrated metabolic process, and identifying the intersections of these pathways, estimating the influence of the growing environment, and determining which compounds are limiting for the resultant compositional profiles is still an area of active investigation (Kambhampati et al. 2021). This work shows that the fatty acid and carbohydrate profile traits are unlinked and can be combined to create soybeans with value-added traits in both meal and oil. We have previously explored the effects of different alleles of the *FAD* genes to obtain the best oil profile, to obtain the highest levels of oleic acid while minimizing linolenic acid, and characterized combinations of the raffinose synthase mutations for the significant reductions of seed RFOs (Held et al. 2019; Sweeney et al. 2017; Hudson and Carrero-Colón 2025; Thapa et al. 2019).

The HO-*rs2rs3* line achieves oleic acid levels consistently above 75%, however linolenic acid levels are between 4–6%. The HOLL-*rs2rs3* lines have > 75% oleic acid, and < 3% linolenic acid, meeting the target (Pham et al. 2012). While loss-of-function alleles for variation in fatty acid content are readily available, there are fewer potential genetic alternatives for the low RFO carbohydrate trait. The most detrimental mutation for *rs2* is the single amino acid deletion allele originally isolated in PI 200508, which has a stronger phenotype than a missense allele identified with reverse genetic approaches (Dierking and Bilyeu 2009a, b). For *rs3*, only two missense mutations exist, and neither has a strong phenotype alone making the isolation of new genetic variants in this gene challenging using forward genetics approaches (Hagely, et al. 2020). Here we combined the PI200508 *rs2* allele with the reverse genetics-isolated G75E *rs3* allele (Thapa et al. 2019). Both the HO-*rs2rs3* and HOLL-*rs2rs3* lines have < 0.1% raffinose and < 0.4% stachyose. Breeding targets to reduce seed RFOs have been defined as less than 0.15% seed mass for raffinose and < 0.54% seed mass for stachyose, and this is designated the ultra-low RFO (uL-RFO) profile (Hagely et al. 2013; Schillinger et al. 2013). Neither the carbohydrate traits nor the fatty acid traits were demonstrated to impact overall protein or oil content, however, this question should be further investigated in high protein or high oil genetic backgrounds. Both the HO-*rs2rs3* and HOLL-*rs2rs3* lines contain > 42% protein on a dry weight basis to meet the minimum acceptable protein meal goals. Additionally, the *rs2rs3* combination has been demonstrated to be less detrimental to seed vigor and germination than other carbohydrate variants in soybean (Dierking and Bilyeu 2009a, b; Lee et al 2023).

The reduction in the non-digestible carbohydrates is significant in the HO-*rs2rs3* and HOLL-*rs2rs3* lines, and meets the threshold for the uL-RFO profile to meet goals for improved digestibility (Parsons et al. 2000). In other studies, a significant increase in sucrose has been observed in *rs2* and *rs2rs3* mutants, which is expected to enhance the metabolizable energy and improve the feeding efficiency of meal including the soybean carbohydrate fraction (Hagely et al. 2020; Bilyeu and Wiebold 2016). Seed sucrose content has been shown to be the most sensitive to environmental factors such as temperature during seed filling, with lower temperatures during seed filling resulting in the highest levels of sucrose (Bilyeu and Wiebold 2016; Kumar et al. 2010; Lee et al. 2023). Here, we did not observe a significant increase in sucrose in the *rs2rs3* mutants in any season, in fact levels of sucrose appear to be reduced in HO and HOLL lines (Fig. 4a, d). As the *rs2* and *rs3* alleles used here are identical to those used in prior studies (Hagely et al. 2020; Kim et al. 2024), we surmise that the differences we have observed may be conferred by the Williams-82 background (from which all alleles used here with the exception of *rs2*_*W331-*_ are derived), possibly also interacting with the environmental variations at this location. The limited increase in sucrose levels in this allele combination is consistent with previous studies in this location and in the Williams82 background (Thapa et al. 2019). This degree of variability in sucrose levels indicates that there is more to understand regarding the effects of growing environment on sucrose levels and that it is likely that there are as-yet uncharacterized and unselected loci to confer elevated sucrose, thereby increasing these soluble carbohydrates for food and feed purposes.

These findings confirm other studies of the combining ability of the HOLL and low/ultralow RFO traits in soybean (Kim et al. 2024) and extend the results to the HO-ultra low-RFO combination. With an eye towards the efficiency of trait stacking, we conclude that in some environments it may be possible to achieve the high oleic, ultra low RFO phenotype with only three genes (*fad2-1a*, *fad2-1b*, and *rs2*) – the smallest number of new alleles combined to achieve the desired levels of oleic, linolenic, and RFOs, but further study will be required to increase sucrose levels.

## Supplementary Information

Below is the link to the electronic supplementary material.

Supplementary file1 (PDF 209 KB)

Supplementary file2 (PDF 5409 KB)

Supplementary file3 (PDF 408 KB)

Supplementary file4 (PDF 331 KB)

## Data Availability

Raw data analyzed here is available from the authors upon reasonable request.
