# Supplementary material for: Combinations of mutations in the raffinose synthase genes and the fatty acid desaturase genes for improvement of soybean oil and meal traits
Source: Mol Breed. 2026 Jan 23;46(2):13. doi: 10.1007/s11032-026-01636-x (PMC12830528; doi:10.1007/s11032-026-01636-x)
Supplement: Supplementary file 1 — Supplementary file1 (PDF 209 KB) [file 11032_2026_1636_MOESM1_ESM.pdf]

**Figure S1. Genotypes and genetic combinations for lines used in this study**

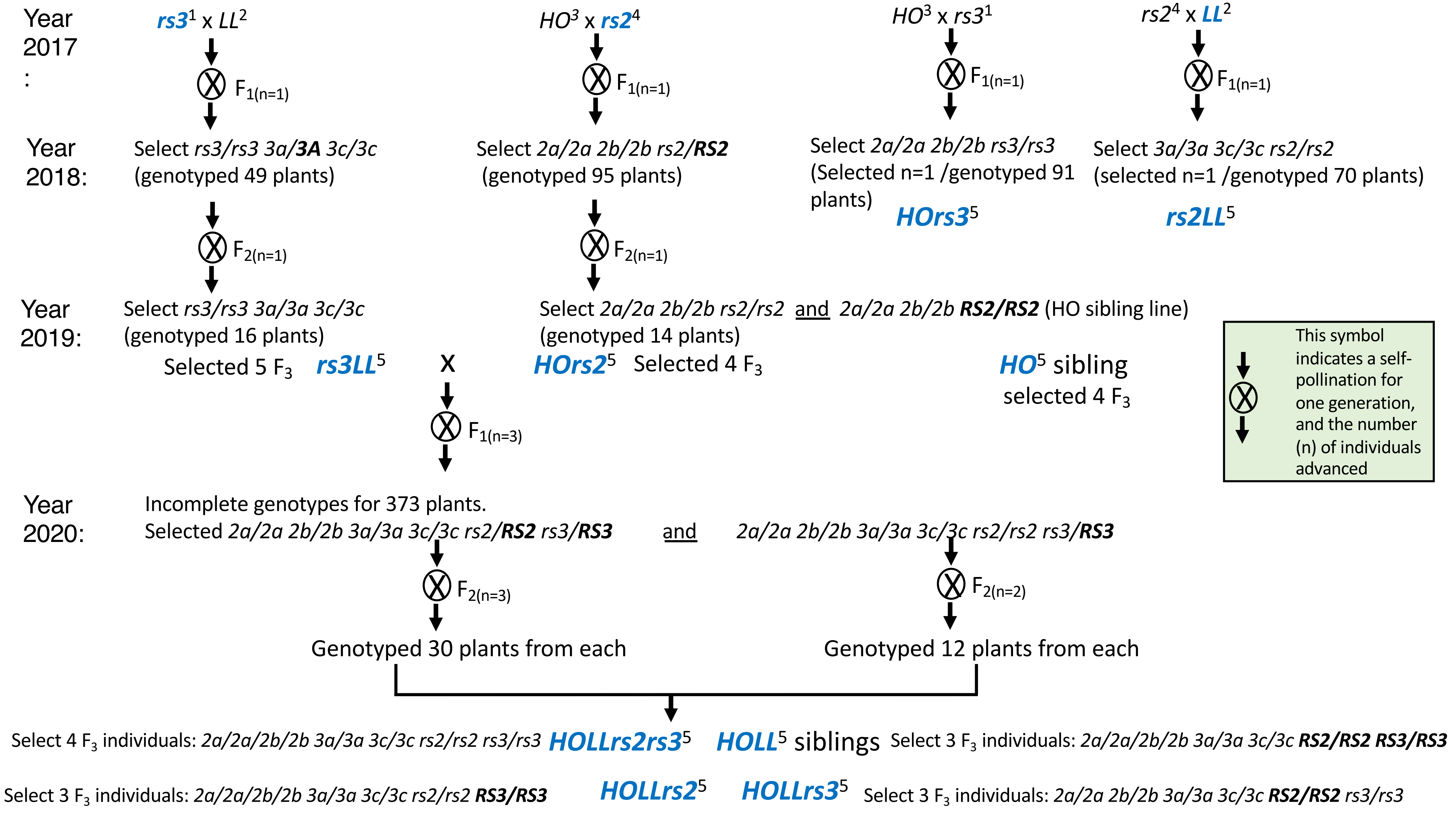


Key: Bold **BLUE** text indicates the genotypes/ lines used for the composition study. After selection by genotyping and verification by phenotyping by GC, the individuals were propagated as self-pollinating inbred lines in composition studies conducted from 2021-2023. When homozygous mutants were not obtained by genotyping the initial $F_2$ population, we advanced one or more heterozygous individuals (wild type allele indicated in bold), allowing it to self pollinate.

1. *rs3* indicates the *rs3*$_{G75E}$ single mutant in W82, genotype is annotated *rs3/rs3*. (Thapa, et al. 2019).
2. *LL* is *fad3a*$_{W81STOP}$ *fad3c*$_{P266S}$ described in Held, et al. (2019). Genotype of the double mutant is annotated *3a/3a 3c/3c.*
3. *HO* is *fad2-1a*$_{W194STOP}$*fad2-1b*$_{P284S}$ from Sweeney, et al., 2017. Genotype of the double mutant is annotated *2a/2a 2b/2b.*
4. KB13-17-#1341 *rs2*$_{W331D}$ from Jo, 2016. Genotype of the double mutant is annotated *rs2/rs2.*
5. This is the first description of these lines.
