# Supplementary figures and images for: Combinations of mutations in the raffinose synthase genes and the fatty acid desaturase genes for improvement of soybean oil and meal traits
Source: Mol Breed. 2026 Jan 23;46(2):13. doi: 10.1007/s11032-026-01636-x (PMC12830528; doi:10.1007/s11032-026-01636-x)

Figure S2. Seed morphology of HO and HOLL combinations

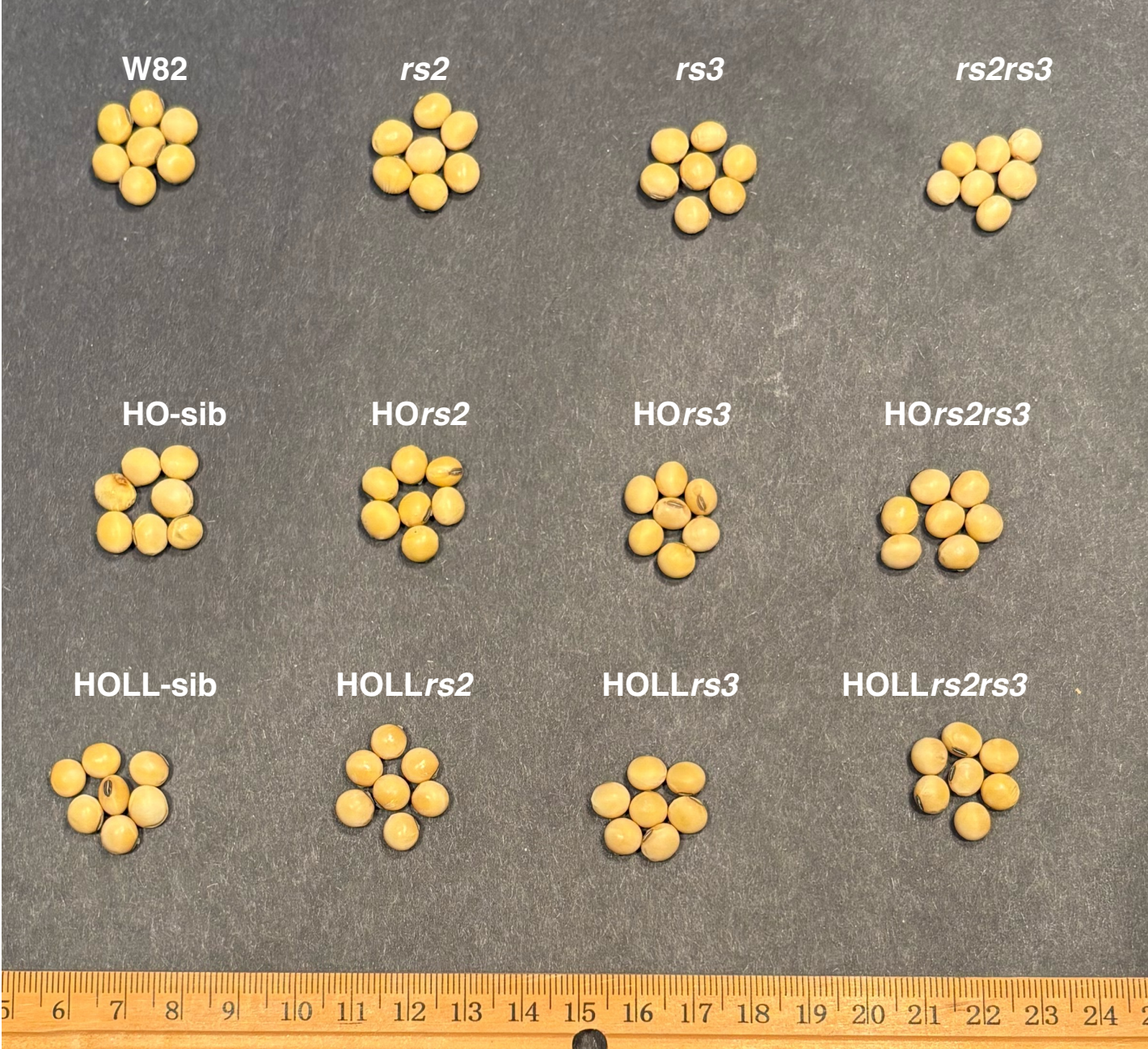

Supplement: Supplementary file 2 — Supplementary file2 (PDF 5409 KB) [file 11032_2026_1636_MOESM2_ESM.pdf]
