# Supplementary material for: Combinations of mutations in the raffinose synthase genes and the fatty acid desaturase genes for improvement of soybean oil and meal traits
Source: Mol Breed. 2026 Jan 23;46(2):13. doi: 10.1007/s11032-026-01636-x (PMC12830528; doi:10.1007/s11032-026-01636-x)
Supplement: Supplementary file 3 — Supplementary file3 (PDF 408 KB) [file 11032_2026_1636_MOESM3_ESM.pdf]

Figure S3. Fatty acids in HO, HO-*rs2*, HO-*rs3*, and HO-*rs2rs3*

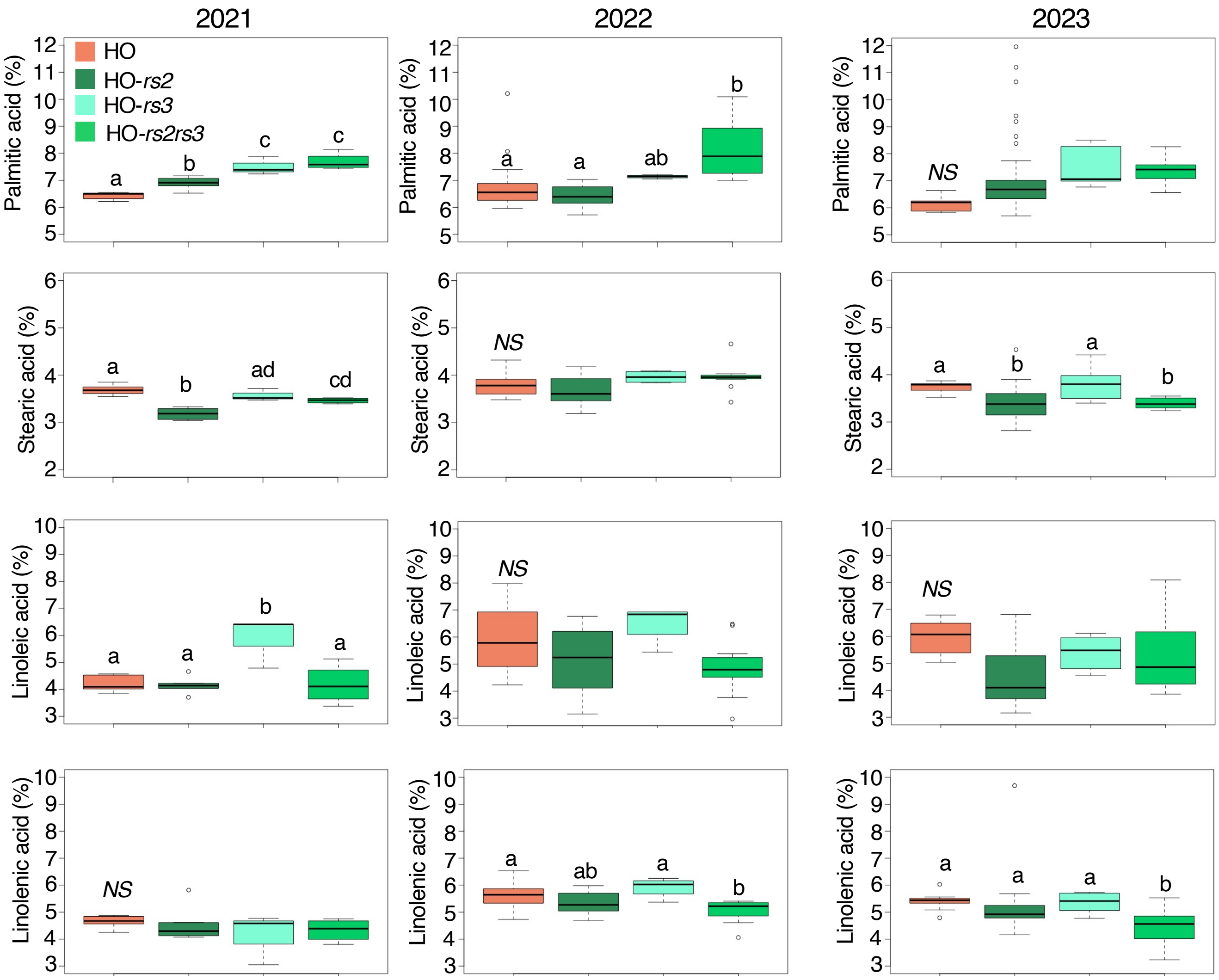

Fig S3 Fatty acids in HO lines carrying combinations of the *rs2* and *rs3* alleles over three growing seasons. Fatty acids are expressed as a percent of total fatty acids. Significance by Tukey HSD at $p < 0.05$.
