# Supplementary material for: Combinations of mutations in the raffinose synthase genes and the fatty acid desaturase genes for improvement of soybean oil and meal traits
Source: Mol Breed. 2026 Jan 23;46(2):13. doi: 10.1007/s11032-026-01636-x (PMC12830528; doi:10.1007/s11032-026-01636-x)
Supplement: Supplementary file 4 — Supplementary file4 (PDF 331 KB) [file 11032_2026_1636_MOESM4_ESM.pdf]

Figure S4 Other Fatty acids in HOLL, HOLL-*rs2*, HOLL-*rs3,* HOLL-*rs2rs3*

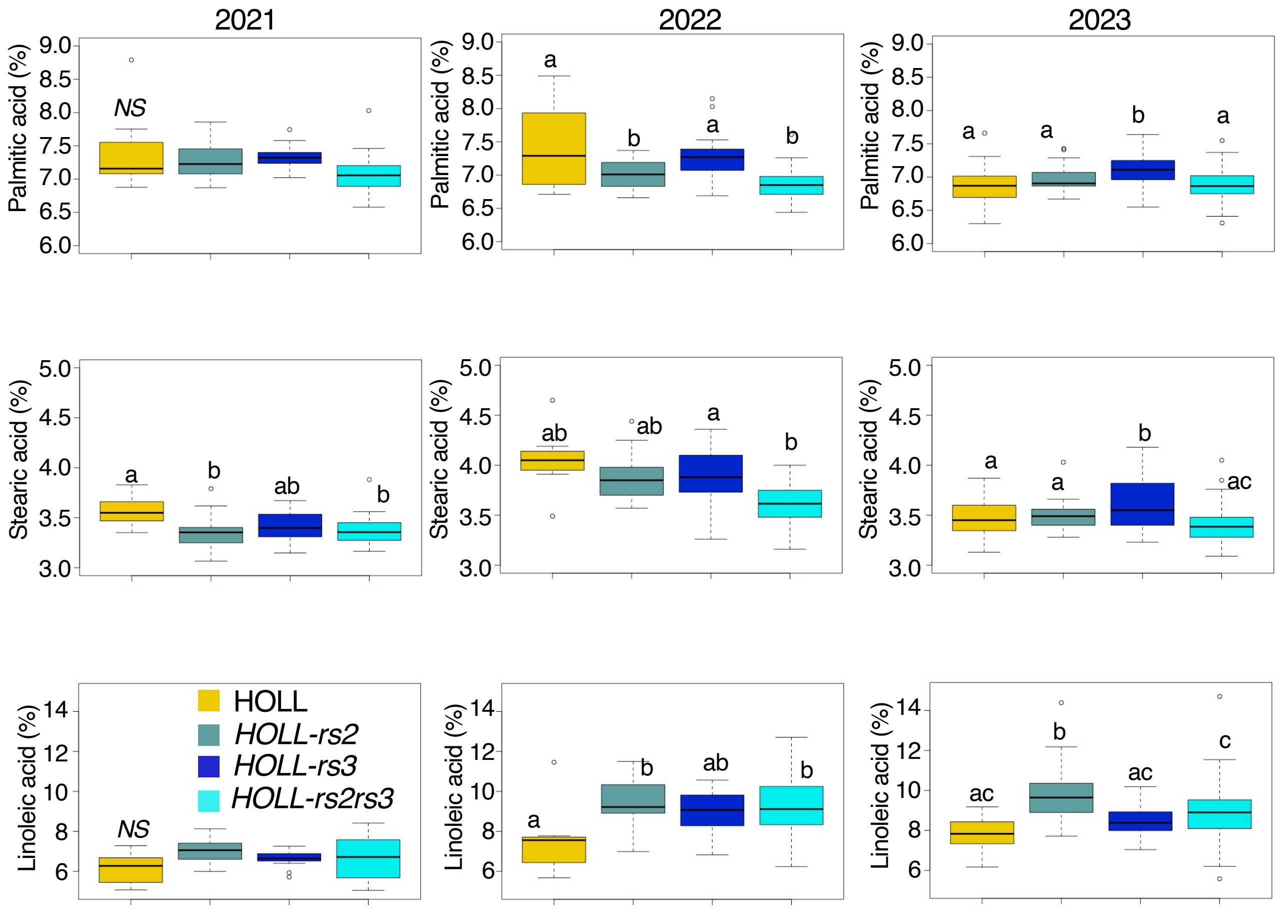

Fig S4 HOLL lines carrying combinations of the *rs2* and *rs3* mutant alleles over three growing seasons. All lines carry *fad2-1a*, *fad2-1b*, *fad3a*, and *fad3c* mutant alleles. Fatty acids are expressed as a percent of total fatty acids.
